# Malignant Acute Disseminated Encephalomyelitis (ADEM) Presenting as a Stroke Alert: A Case Report

**DOI:** 10.7759/cureus.24961

**Published:** 2022-05-13

**Authors:** Madeline J Hooper, Joshua A Kalter, Nicholas S Imperato, Marna R Greenberg

**Affiliations:** 1 Department of Emergency and Hospital Medicine, University of South Florida Morsani College of Medicine/Lehigh Valley Health Network Campus, Allentown, USA

**Keywords:** ring-enhancing lesions, intravenous immunoglobulins (ivig), multiple sclerosis and other demyelinating disorders, post-infectious adem, plex, acute disseminated encephalomyelitis (adem)

## Abstract

Acute disseminated encephalomyelitis (ADEM) is a rare illness. Generally characterized by encephalopathy and non-specific, heterogeneous neurological deficits depending on the location of the demyelinated lesions, ADEM is considered a clinical diagnosis with radiological findings that may or may not have supportive features based on the temporal relationship of an inciting factor and symptom onset. Even rarer, hyperacute or malignant ADEM can be defined by rapid symptom onset followed by catastrophic brain edema and its sequelae. We present a case of a patient who presented with an acute stroke with activation of a rapid sequence care pathway (stroke alert protocol) to mobilize resources that could expedite his care to determine eligibility for thrombolysis. ADEM was the definitive diagnosis with a subsequent rapid and treatment-refractory decline.

## Introduction

It is estimated that one in 125,000-250,000 individuals is affected by acute disseminated encephalomyelitis (ADEM) each year [1]. A rare illness, ADEM is generally considered a clinical diagnosis with radiological findings that may or may not have supportive features based on the temporal relationship of an inciting factor and symptom onset [2]. Many times, triggered by inflammation secondary to infection or vaccination, ADEM is a demyelinating disease of the central nervous system most often seen in children and young adults with potentially severe neurologic sequelae [2-3]. While antecedent infections are not necessary, it has been noted that between 50% and 75% of ADEM cases were preceded by either infection or vaccination [3]. Historically, the measles virus has been known to induce this disease in approximately one per 1,000 cases while ADEM may occur approximately once in every 10,000 varicella vaccinations administered [4]. In the current coronavirus disease-19 (COVID-19) pandemic, cases of ADEM have been identified following severe acute respiratory syndrome coronavirus 2 (SARS-CoV-2) infection [5-6]. We present a case of a patient who presented with an acute stroke with activation of a rapid sequence care pathway (stroke alert protocol) to mobilize resources that could expedite his care to determine eligibility for thrombolysis. ADEM was the definitive diagnosis with a subsequent rapid and treatment-refractory decline.

## Case presentation

A 21-year-old male presented to the emergency department (ED) in eastern Pennsylvania with a pre-hospital stroke alert with left-sided weakness, mumbling speech, and confusion. Two months prior, he was noted to have sudden onset confusion and slowed speech at a party in the northwestern USA. At that time, he was admitted to a hospital, where a complete workup, including a brain MRI, identified approximately 25 ring-enhancing lesions. Additional workup with CSF studies was negative, and the patient was diagnosed with ADEM. He was treated with IV methylprednisolone and was discharged to inpatient rehabilitation on a prolonged oral steroid taper. Six weeks after the initial presentation, he had moved back to Pennsylvania, where he had continued to improve. Due to an acute flare in neurological symptoms, he sought emergency care.

**Figure 1:**
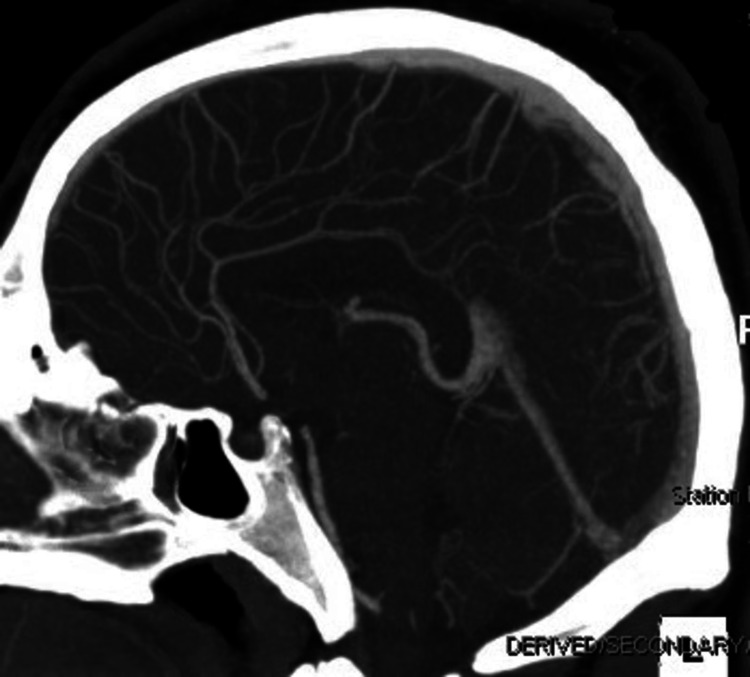
CT angiogram showing no acute large vessel occlusions

**Figure 2:**
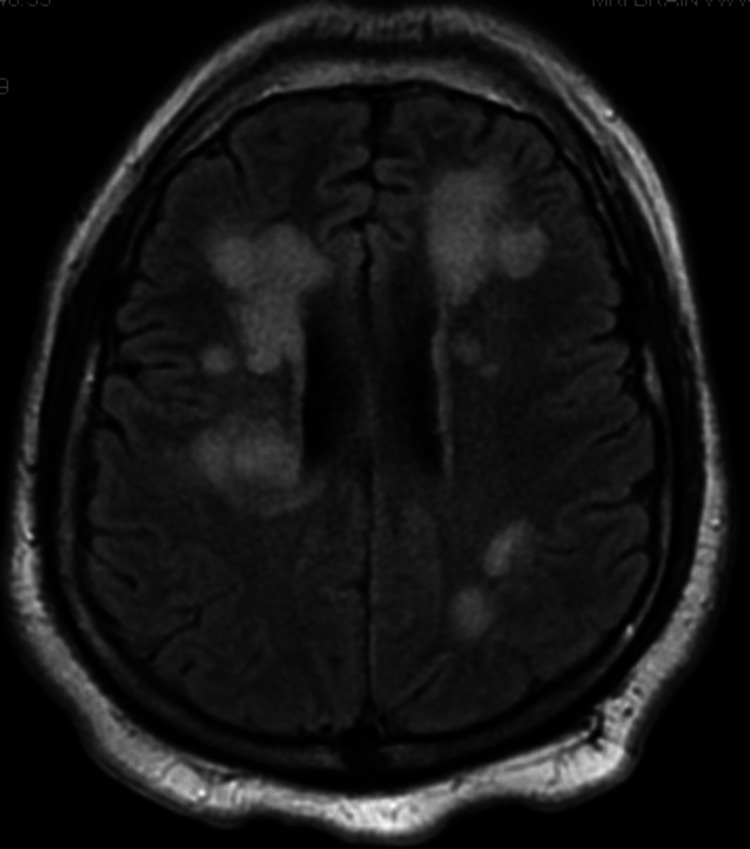
Initial MRI of the brain taken early in the patient’s hospital stay Multiple areas of hyperintensity were noted but without significant mass effect.

On arrival at the ED, the patient displayed mild left-sided neglect, with only minimal movement of his left arm and left leg and a right gaze preference. The patient was awake, alert, and oriented only to self and time with notable slurred speech and an asymmetric smile. He was afebrile with a heart rate of 80 beats per minute, blood pressure of 163/92 mmHg, and oxygen saturation of 97%. Due to the focal neurological deficit and the potential for cerebrovascular accident (CVA), a "stroke alert" pathway was initiated to mobilize resources, including rapid neurologist response and imaging that expedited his care to determine eligibility for thrombolysis. A CT angiogram showed no acute large vessel occlusion or signs of acute bleed (Figure 1). Complete blood count was within normal limits, a comprehensive metabolic panel showed mildly elevated alanine aminotransferase (ALT) at 104 U/L (reference: < 56 U/L), and urine drug screen was positive for cannabinoids. He was given 1 g IV levetiracetam for concern for seizures, and MRI brain without contrast showed multiple prominent areas of T2/fluid-attenuated inversion recovery (FLAIR) hyperintensity involving the periventricular, subcortical, and juxtacortical white matter bilaterally as well as the pons and bilateral middle cerebellar peduncles. Some lesions demonstrate mild, partial diffusion restriction. There was no substantial mass effect (Figure 2).

Lumbar puncture was performed, and CSF analysis (Table 1) showed elevated protein, red blood cells, and white blood cells with lymphocytic predominance and was positive for oligoclonal bands of immunoglobulin G (IgG). CSF cytology showed no immunophenotypic evidence of a clonal B- or T-cell population. A CSF meningitis/encephalitis panel (polymerase chain reaction (PCR)) was negative for all major bacterial, viral, or fungal etiologies and cultures did not display any growth. Further blood cultures were negative for toxoplasmosis, HIV, and tuberculosis. CSF paraneoplastic and encephalitis panels were negative. Additionally, the patient was tested on three separate occasions throughout his hospital stay for SARS-CoV-2 (COVID-19), via PCR tests, all of which were negative.

**Table 1:** CSF analysis of patient at admission

	CSF Studies at Admission	Reference
Glucose	60 mg/dL	40-70 mg/dL
Protein	71 mg/dL	15-45 mg/dL
RBC	29,386/cm^3^	0-5 /cm^3^
WBC	132/cm^3^	0-5 /cm^3^
Neutrophils	14%	0-2%
Lymphocytes	83%	63-99%
Monocytes	0%	3-37%

Serum thyroid-stimulating hormone (TSH), B12, ammonia, C-reactive protein, erythrocyte sedimentation rate, C3 and C4 complements, and angiotensin-1-converting enzyme were within normal limits. Vitamin D, 25-OH was decreased at 10 ng/mL (reference: 30-100 ng/mL). Serum rheumatoid factor, anti-dsDNA, ANA, SSA, SSB, SM/RNP, Sm, SCL-70, proteinase 3, myeloperoxidase, cyclic citrullinated peptide, MOG, and aquaporin 4 receptor antibodies were all negative.

Over the next 24 hours, the patient began to rapidly decompensate, losing the ability to follow commands. He was then started on 1 gram IV solumedrol. A repeat head CT did not identify any specific changes that could correlate to the patient’s declining status. Continuous electroencephalography (EEG) monitoring showed right frontal rhythmic delta activity (RDA) with stim-induced left frontal RDA and focal delta over the right, which resolved, as well as diffuse theta activity. On hospital day 4, the patient’s EEG began to show diffuse slowing, right greater than left RDA with associated sharp waves and prolonged runs of bilateral RDA at 1-2 Hz. This finding was consistent with diffuse encephalopathy and a lowered seizure threshold.

**Figure 3:**
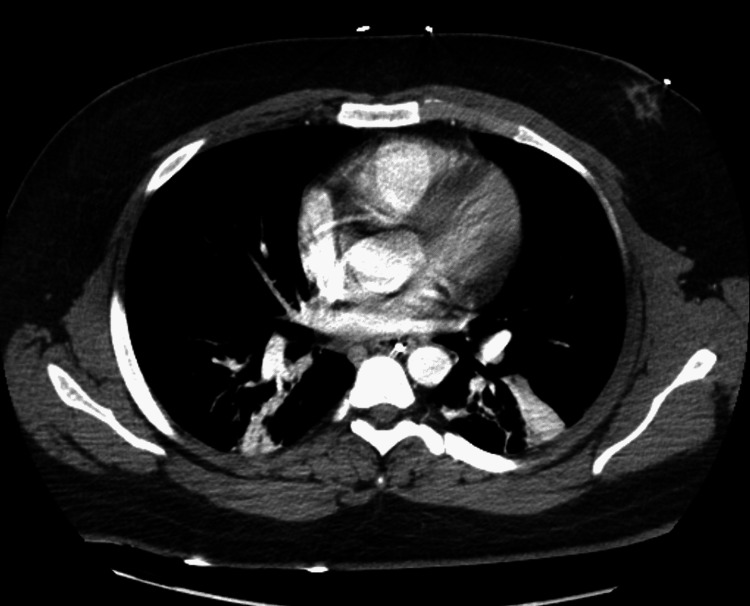
CT of the chest identifying scattered patchy ground-glass opacities in the posterior portion of the right middle lobe

**Figure 4:**
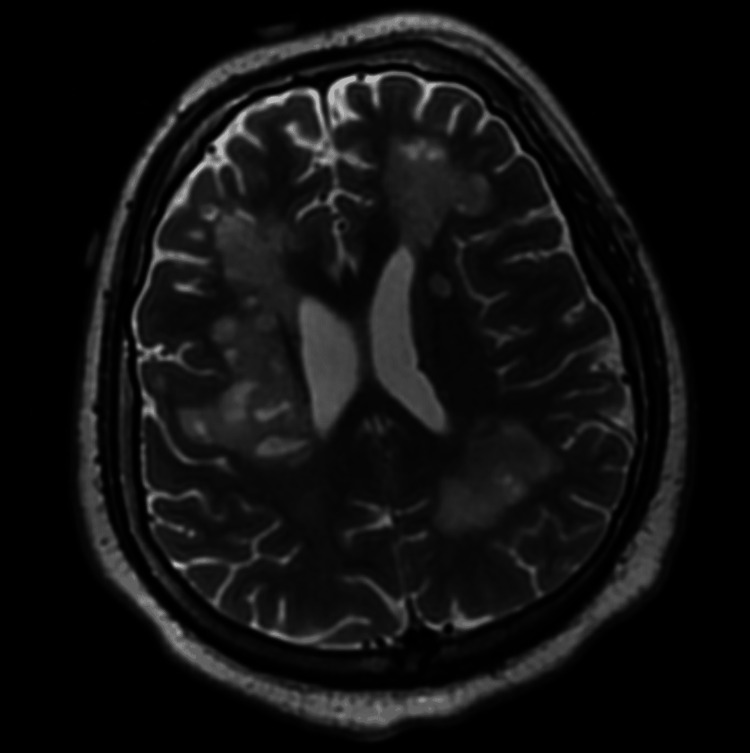
MRI of the brain taken later in the patient’s hospital stay, identifying an increase in the size and quantity of lesions

The next day, the patient began contracting his upper extremities bilaterally, and his vitals became unstable, with a respiratory rate of 48, heart rate of 170 beats per minute, blood pressure of 190/90 mmHg, and temperature of 104.3°F. He became increasingly hypoxic despite increasing oxygen administration, requiring intubation and transfer to the neuroscience intensive care unit (NSICU). A CT scan of the patient’s chest showed bilateral lower lobe airspace disease and patchy right upper and middle lobe ground-glass nodular opacities (Figure 3). He was then started on empiric vancomycin and cefepime for pneumonia. A repeat MRI of the brain identified a significant increase in the size and quantity of lesions, with new supratentorial lesions noted (Figure 4).

After this point, the patient only displayed minimal clinical improvement with a five-day solumedrol pulse and was then started on a prolonged course of 40 mg prednisone, administered via nasogastric tube. The patient’s lack of improvement led to the initiation of plasma exchange (PLEX), where he received five cycles, before restarting a five-day methylprednisone pulse. Neurosurgery was consulted to perform a brain biopsy, which did not yield a definitive diagnosis but was consistent with demyelination. Specifically, the pathology interpretation of the biopsy reported benign brain parenchyma with several collections of macrophages in a background of reactive gliosis. Luxol fast blue (LFB)/periodic acid-Schiff (PAS) stained sections demonstrated multifocal areas of pallor consistent with demyelination and immunohistochemistry confirmed the macrophagic infiltrate with collections of CD68 positive cells. GFAP stained sections highlighted the reactive gliosis background. CD3 stained sections revealed scattered positive T-lymphocytes.

One month after initial hospitalization, the patient became acutely hypoxic before going into cardiac arrest. The patient was given IV tPA, as he was believed to have had a pulmonary embolism. Resuscitation efforts attained multiple brief periods of return of spontaneous circulation (ROSC), but, after discussion with family, they were stopped.

## Discussion

ADEM can manifest as a new neurological deficit, and in our case, malignant ADEM was based on the patient's rapid and treatment-refractory decline. Possible inciting factors could include an unreported febrile infection, a previous asymptomatic COVID-19 infection, or recreational drug use.

Characterized by encephalopathy and non-specific, heterogeneous neurological deficits depending on the location of the demyelinated lesions, ADEM is a clinical diagnosis with radiological findings that may or may not have supportive features based on the temporal relationship of an inciting factor and symptom onset [3]. Occurring in only 2% of cases, hyperacute or malignant ADEM is defined by rapid symptom onset followed by catastrophic brain edema and its sequelae [2-3,7].

Although considered an autoimmune disease, the exact pathogenesis of ADEM is unknown. During the acute phase of ADEM, pro-inflammatory cytokines and antibodies targeting gangliosides are suspected to cause demyelinating injury to white matter in the brain and spinal cord. In ADEM, T2-weighted and FLAIR MR images of the brain are significant for multiple, bilateral, and poorly defined lesions in deep cortical and subcortical areas, including the thalami and basal ganglia [2-3]. Large, tumefactive lesions can also affect the spinal cord [8-9]. Notably, while ADEM is typically monophasic because it is precipitated by a specific event, contrast-enhanced MR images will often identify new lesions within the first 30 days following the initial attack [10]. This observation may be due to ADEM-specific changes that delay cytokine and antibody clearance, thus facilitating prolonged autoimmune injury. Moreover, cases have also been reported in which imaging findings are delayed by more than one month after symptom onset [11]. CSF changes in ADEM frequently demonstrate increased pressure, lymphocytic pleocytosis, and increased protein levels.

First-line treatment for ADEM involves IV steroids during the acute phase, followed by an oral steroid taper over the subsequent three to six weeks. Beyond this, plasmapheresis and IV immunoglobulin (IVIG) have been successfully used to treat steroid-resistant cases. In the case review published by Keegan et al., plasmapheresis resulted in clinical improvement in 50% of patients with steroid-refractory ADEM, even when the therapy was initiated more than 60 days after symptom onset [12]. While there has been no randomized controlled trial to evaluate the effectiveness of first-line, high-dose steroids or second-line plasmapheresis in ADEM, excellent therapy response has been reported for both regimens [13-14]. Despite this promising evidence, our patient failed to improve even after five cycles of plasmapheresis on top of high-dose IV corticosteroids. Cyclophosphamide and mitoxantrone are used for severe immunosuppression, but these interventions were not pursued in our case [3]. Overall, 50% of patients recover completely from ADEM and early initiation of treatment and preserved neurological functioning are associated with improved prognosis; mortality is between 10% and 30% [7].

As symptoms can evolve within three months following an ADEM attack, this patient’s presentation after six weeks of treatment is best characterized as corticosteroid-refractory rather than recurrent or multiphasic. Given that MRI findings can worsen during the months following symptom onset, the fact that our patient’s brain imaging did not improve between his initial episode and his admission to our institution is also consistent with the typical course of ADEM. Still, the severity of his illness is significant within the context of an unknown inciting factor and warrants further investigation.

While the patient tested negative for COVID-19 during his admission, there were no records officially documenting results of his COVID status from the hospital in Washington, which does not exclude the fact that he had an asymptomatic case at some point before symptomology. The neuropathology of COVID-19 is varied, and reports have connected positive serology with demyelination, encephalitis, stroke, and neuropathy [6]. The case report by Parsons et al. described ADEM as a parainfectious process secondary to COVID-19 infection in a 51-year-old woman that was responsive to IV steroids and IVIG over several weeks of treatment [5]. In their systematic review, Montalvan et al. noted more evidence was needed to connect ADEM to any human coronavirus infection [6]. Nonetheless, given the concern that SARS-CoV-2 may act as a neuropathogen, it would appear to be a pertinent differential diagnosis.

Because of its rarity, malignant ADEM is not well-described in the literature [7]. Our patient’s presentation with acute left-sided weakness, mumbling speech, and confusion that was concerning for acute CVA. This was an unusual case particularly for the acute care clinicians (neurologists and emergency physicians) to determine the source of the patient’s neurological deficit. The differential of ADEM is challenging to consider in the context of the time-sensitivity required for CVA interventions. There are similarities and contrasts between the two diagnoses relevant to acute care (Table 2) that make the differential determination challenging. In our case, the patient’s acute care was refractory to steroids and plasmapheresis and ultimately progressed to decorticate posturing, respiratory failure, and death is consistent with this devastating variant of ADEM.

**Table 2:** Comparison and contrast to consider in the acute presentation of cerebrovascular accident (CVA) and acute disseminating encephalomyelitis (ADEM)

CVA	ADEM
Prevalence not uncommon	Prevalence rare
Acute focal neurological signs are a primary indicator for further evaluation	May present with acute focal neurological signs
Etiology from a cerebral blocked artery (ischemic) or bleeding (hemorrhagic)	Triggered by inflammation secondary to infection or vaccination
Definitive ischemic or hemorrhagic stroke is usually a monophasic course but maybe mimicked by transient ischemic attacks	Usually a monophasic course, maybe multiphasic making it more difficult to differentiate from other diagnoses (multiple sclerosis, transient ischemic attacks)
Computed tomography angiography (CTA) findings consistent with cerebral vessel occlusion or hemorrhage	CTA has no cerebral occlusions or hemorrhage
Although conventional MRI sequences most often do not show evidence of stroke in the acute phase, conventional MRI may show signs of intravascular thrombi, such as the absence of flow void on T2-WI, vascular hyperintensity on fluid-attenuated inversion recovery (FLAIR), and hypointense vascular sign on gradient-recalled echo (GRE) sequence. Hemorrhage can be detected	T2-weighted and FLAIR MR images of the brain are significant for multiple, bilateral, and poorly defined lesions in deep cortical and subcortical areas, including the thalami and basal ganglia
Treatment may include thrombolysis or clot retrieval or medications for hemorrhage	The primary treatment is steroids

## Conclusions

This case helps clinicians broaden their differential for patients presenting with acute neurological deficits. Additionally, it adds to the paucity of discussion in acute care literature on hyperacute or malignant ADEM. While identifying the causative etiology of ADEM does not impact treatment, it is important to consider the increasing number of reports connecting COVID-19 infection to acute and chronic neurological disorders. Furthermore, our patient’s failure of both high-dose steroids and plasmapheresis underscores the importance of early, aggressive treatment initiation and recognition that second-line therapies may need to be added on expeditiously to prevent irreversible neurological damage.
